# Porous Si/Fe_2_O_3_ Dual Network Anode for Lithium–Ion Battery Application

**DOI:** 10.3390/nano10122331

**Published:** 2020-11-25

**Authors:** Yanxu Chen, Yajing Yan, Xiaoli Liu, Yan Zhao, Xiaoyu Wu, Jun Zhou, Zhifeng Wang

**Affiliations:** 1 School of Materials Science and Engineering, Hebei University of Technology, Tianjin 300401, China; yxchen116@163.com (Y.C.); 201921801024@stu.hebut.edu.cn (Y.Y.); xywuhebut@163.com (X.W.); 2 Key Laboratory for New Type of Functional Materials in Hebei Province, Hebei University of Technology, Tianjin 300401, China; 3 School of Materials Science and Engineering, Hebei University of Science & Technology, Shijiazhuang 050018, China; iven308@126.com

**Keywords:** dealloying, porous, dual network, lithium–ion battery, anode

## Abstract

Benefiting from ultra-high theoretical capacity, silicon (Si) is popular for use in energy storage fields as a Li–ion battery anode material because of its high-performance. However, a serious volume variation happens towards Si anodes in the lithiation/delithiation process, triggering the pulverization of Si and a fast decay in its capacity, which greatly limits its commercial application. In our study, a porous Si/Fe_2_O_3_ dual network anode was fabricated using the melt-spinning, ball-milling and dealloying method. The anode material shows good electrochemical performance, delivering a reversible capacity of 697.2 mAh g^−1^ at 200 mA g^−1^ after 100 cycles. The high Li storage property is ascribed to the rich mesoporous distribution of the dual network structure, which may adapt the volume variation of the material during the lithiation/delithiation process, shorten the Li–ion diffusion distance and improve the electron transport speed. This study offers a new idea for developing natural ferrosilicon ores into the porous Si-based materials and may prompt the development of natural ores in energy storage fields.

## 1. Introduction

With the fast development of the market economy, the requirements for energy storage devices are constantly improving [1,2,3]. Lithium–ion batteries (LIBs) are identified as attractive energy maintaining devices because of their high specific energy, long service life and excellent environmental compatibility [4,5,6]. At present, the low theoretical capacity of the graphite anode restricts its commercial application in the high-end market [7,8]. To enhance the properties of LIBs further, it is necessary to develop new anodes with high performance. Silicon (Si) is considered to be one of the potential candidates to substitute conventional carbon anode for next-generation LIBs on account of the characteristics of extremely high theoretical mass-specific capacity (4200 mAh g^−1^), low working voltage and high natural abundance [9,10]. The main obstacle to its practical application is the tremendous volume variation (≈420%) during repeated lithiation/delithiation procedures [11], bring about pulverization, shedding of active materials and rapid decay of capacity [12,13,14]. To accommodate the volume variation of Si and boost the electrochemical properties of LIBs, some strategies have been proposed by scholars from various countries. Firstly, once the Si materials were produced into different nanomaterials, such as nanoparticles, nanotubes, nanowires, nano hollow structures and so on [15,16,17], they can provide shorter transmission paths and reduce the diffusion distance of Li^+^. Secondly, many Si nanostructures may provide sufficient space to suppress the volume variation and capacity attenuation [18,19,20]. At present, the traditional methods of preparing Si-based nanostructured materials mainly include chemical vapor deposition, high-temperature laser evaporation, self-assembly growth method and so on [21,22]. Furthermore, the formation of composites between Si and metal oxides (relatively low theoretical specific capacity) can also alleviate the volume change and structural damage of Si anodes to some extent, inducing an improvement in cycling stability [23]. David Zitoun et al. [24] synthesized Si/γ-Fe_2_O_3_ as an anode material for LIBs by successive organometallic decomposition of pentacarbonyl-iron on Si nanomaterials subsequently by redox reactions, demonstrating a high reversible capacity of 2600 mAh g^−1^; however, the above method seems difficult to satisfy the demands of industrial application because of its complex preparation process, strict preparation conditions and high cost both in materials and equipment.

In the current work, we propose a new process to fabricate porous Si/Fe_2_O_3_ dual-network composite derived from natural ferrosilicon ores using the melt-spinning, ball-milling and dealloying process, in which the dual network structure, with abundant mesopores, contains the low-capacity Fe_2_O_3_ network and the high-capacity Si network. In addition, natural ferrosilicon ores are abundant in reserves and are used as deoxidizers in the steelmaking industry. They are used to synthesize anode materials of LIBs in this study, which greatly reduces the cost of raw materials, meaning that ferrosilicon ores can also be used as a cheap and sufficient resource for large-scale preparation of Si-based anode materials. The strategy also provides an idea for the preparation of low-cost Si-based anode materials.

## 2. Materials and Methods

**Figure 1:**
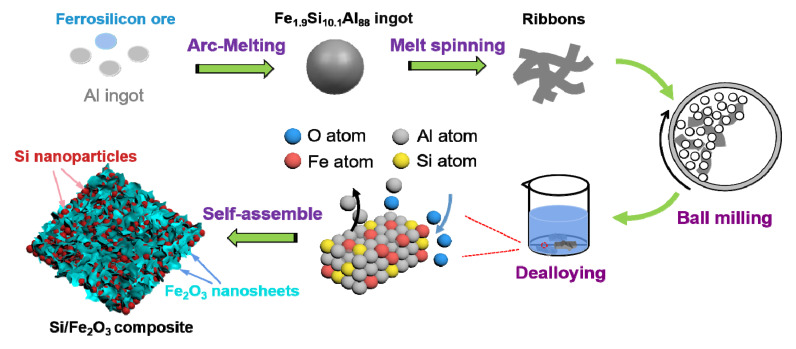
Schematic diagram presenting the fabrication route of Si/Fe_2_O_3_.

The typical synthesis route is shown in Figure 1. Firstly, the arc-melting method [25] was used to prepare the master alloy ingot. Ferrosilicon ore (Fe: 27.15 wt.%, Si: 72.36 wt.%, total of other associated elements Mn, C, S, P, etc.: 0.49 wt.%) and aluminum ingots (99.99 wt.%) were produced into a Fe_1.9_Si_10.1_Al_88_ alloy ingot by high-temperature electric arcs. Then, Fe_1.9_Si_10.1_Al_88_ ribbons were obtained using the melt-spinning process [26]. In this situation, the melts were sprayed onto a water-cooled Cu roller with a rotate speed of 1800 r/min to produce ribbons tens of centimeters long, 20 microns thick and 3 mm wide. A 2.0 g ribbon was placed in a ball mill tank with stainless steel balls, where the ratio of grinding media to material was 20:1. To prevent oxidation of the sample, n-heptane was added into the tank and the liquid level was ensured to exceed the sample. Three kinds of powder precursors were obtained by ball-milling the ribbons for 24–72 h at a rotation speed of 600 r/min at ambient temperature. The samples were rinsed with anhydrous ethanol to remove n-heptane and dehydrated in a vacuum drying box at 60 °C for 12 h to obtain the BM-24, BM-48 and BM-72 samples, respectively. Then, the above samples were dealloyed in 1.25 M NaOH solution for 4 h [27,28]. After washing in ethyl alcohol and drying in vacuum drying box at 60 °C for 12 h, the dealloyed products of BM-24-4, BM-48-4 and BM-72-4 were finally synthesized. In particular, the above process can be improved by a scale-up continuous melt-spinning process [27]. Tens of kilograms of alloy ribbons with a large output can be prepared for each furnace, inducing a relatively low cost per cell. As a result, all the processes are suitable for mass production or can be produced in batches. In addition, the dealloying process is free of costly reagents and free of solutions that could lead to serious environmental pollution, which provides many advantages, such as convenient operation and low environmental pollution.

The phase composition of the samples was analyzed by X-ray diffraction (XRD, Bruker D8-Discover, Karlsruhe, Germany). The valence state of products was studied by X-ray photoelectron spectroscopy (XPS, V-Sorb 2800P, Beijing, China). Raman spectra were tested via the Reflex machine. Scanning electron microscope (SEM, JSM-7100F, Tokyo, Japan) and transmission electron microscope (TEM, JEM-2100F, Tokyo, Japan) were used to observe the microstructure. The Brunauer–Emmett–Teller (BET) method was used to analyze the specific surface and the Barrett–Joyner–Halenda (BJH) method was employed to determine the pore size distribution.

Si/Fe_2_O_3_ material, carboxymethyl cellulose (CMC) and conductive agent (Super P) (8:1:1, mass ratio) were mixed in deionized water to create a slurry. The slurry was smeared onto the surface of the Cu current collector (Cu foil) and dehydrated in a vacuum furnace at 60 °C for 12 h and then cut into discs of 10 mm in diameter to make a working anode. The mass loading of the anode was in the range of 0.89–1.05 mg/cm^2^. In a glove box with an argon environment (H_2_O/O_2_, less than 0.01 PPM), the anode, the lithium tablet cathode, the Celgard 2320 diaphragm and the electrolyte (1 M LiPF_6_ dissolved in EC/DEC, 1:1 in volume ratio) were encapsulated in a CR2032 coin battery case. The electrochemical impedance spectroscopy (EIS) and cyclic voltammetry (CV) curves of the cell were detected through an electrochemical workstation (CHI760E) with a test scan speed of 0.1 mV s^−1^ in 0.01–3 V. Galvanostatic charge/discharge curves were monitored on a newway battery tester in the range of 0.01–3 V (vs. Li^+^/Li).

## 3. Results and Discussion

**Figure 2:**
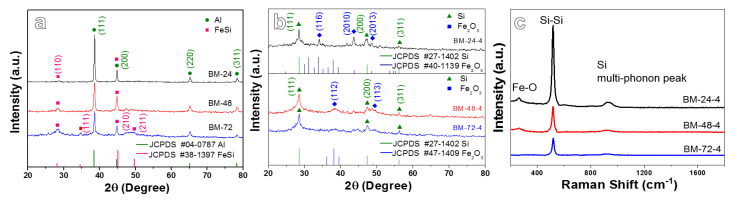
(**a**) XRD patterns of the ball-milled BM-24, BM-48, and BM-72 samples, (**b**) XRD patterns and (**c**) Raman spectrum of the dealloyed BM-24-4, BM-48-4 and BM-72-4 samples.

XRD results of the as-obtained ball-milled samples and the dealloyed samples are presented in Figure 2a,b, respectively. After ball-milling for a different time, the samples present strong diffraction peaks at 38.5°, 44.7°, 65.1° and 78.2°, corresponding to (111), (200), (220) and (311) lattice planes of crystal Al (JCPDS No.04-0787), respectively. While the peaks at 28.1°, 34.6°, 45.1° and 49.7° are consistent with (110), (111), (210) and (211) lattice planes of FeSi phase (JCPDS No.38–1397), respectively [29,30,31]. With the extension of the ball-milling time, a part of the Al elements may dissolve into the FeSi phase, inducing a broader peak. In addition, the relative intensity of the FeSi diffraction peak increases gradually while that of the Al peak declines, indicating that the elemental distribution has greatly changed, as shown in Figure 2a. No diffraction peak of Al can be found in the dealloyed materials (Figure 2b), indicating that the most of Al elements have been leached out. In this situation, Fe elements are oxidized concurrently in the dealloying process by oxygen dissolving in corrosion liquid, generating Fe_2_O_3_. Besides the diffraction peak of Fe_2_O_3_, diffraction peaks of (111), (200) and (311) crystal planes of Si phases are also observed [32,33,34]. The above results confirm that the as-synthesized dealloying product is mainly composed of Si and Fe_2_O_3_ phases. 

Figure 2c displays the Raman spectra of the dealloyed products, the peaks observed at 520 cm^−1^ correspond to Si-Si bonds [35], while the peaks at about 293 cm^−1^ relate to the Fe-O bonds (Fe_2_O_3_) [36]. Furthermore, the peaks in the range of 900–1000 cm^−1^ are in accord with the multi-phonon peaks of Si [37]. The Raman data indicate that the products mainly contain two components of Fe oxides and Si, which is compatible with the XRD result. The inductively coupled plasma (ICP) result shows that the weight ratio of Si, Fe_2_O_3_ and residual Al in the BM-24-4, BM-48-4 and BM-72-4 samples are about 83.7:7.4:8.9, 75.1:19.4:5.5 and 63.2:32.7:4.1, respectively. This indicates that the ball-milling process affects the elemental distribution of the precursor and thus influences the composition ratio of dealloyed products.

**Figure 3:**
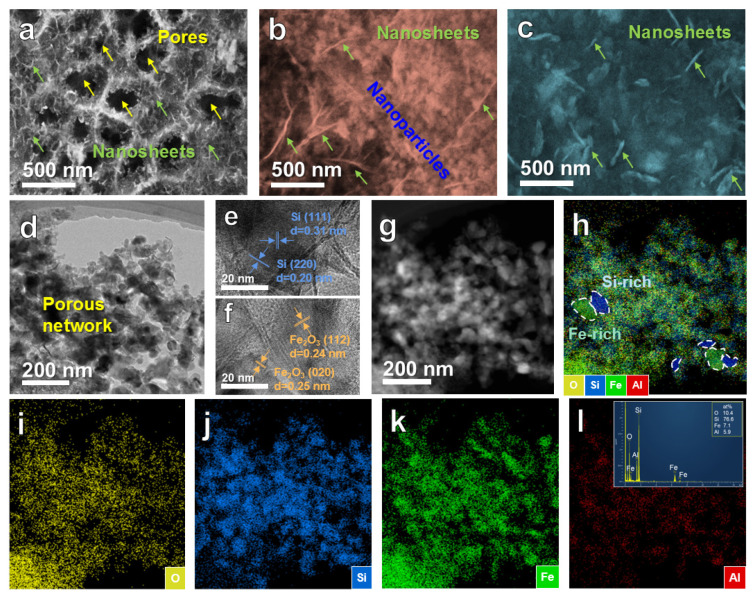
(**a**–**c**) SEM images of the dealloyed BM-24-4, BM-48-4 and BM-72-4 samples; (**d**–**f**) TEM and high-resolution TEM (HRTEM) images, and (**g**–**l**) elemental mapping of the BM-48-4 sample.

The SEM images of the dealloyed products (BM-24-4, BM-48-4 and BM-72-4) are displayed in Figure 3a–c. BM-24-4 presents a typical network-like structure consisting of ligaments and pores. It can be observed that the surface of the ligaments is loaded with nanosheets. A large number of nanosheets with a length of ~140 nm are connected together, but they block the pores of the network, which is not conducive to the passage of lithium ions during charging and discharging process. Figure 3b presents the dual network structure of BM-48-4, which is composed of the nanoparticles (diameter: ≤50 nm) accumulation network and the nanosheets (length of ~500 nm) network. The nanoparticles fill the interspace among the nanosheets network, improving the utilization rate of space. The two sets of networks interpenetrate each other to form a double network structure, which is beneficial to buffer the volume variation in the repeated charging/discharging procedure. Figure 3c shows the microstructure of the BM-72-4 sample. It can be seen that the product is composed of a blocky structure (accumulated by coarsened nanoparticles) and the thick nanosheets network, showing a composite with poor porous structure.

The microstructure of the optimal BM-48-4 sample was further analyzed by TEM. The interlacing distribution of nanoparticles and finely fragmented nanosheets demonstrates that the nanoporous structure is formed across the composite as shown in Figure 3d. The measured crystal interplanar distance of 0.31 and 0.20 nm shown in Figure 3e corresponds to (111) and (220) crystal planes of Si, respectively. While the interplanar distance of 0.24 and 0.25 nm marked in Figure 3f corresponds to (112) and (020) crystal planes of Fe_2_O_3_. These results indicate the co-presence of Si and Fe_2_O_3_, which are consistent with XRD results. The elemental mapping of BM-48-4 shown in Figure 3g–l reveals that Si, Fe, O and a small amount of residual Al elements were distributed in the sample. The energy dispersive X-ray (EDX) spectrum (the insert in Figure 3l) reveals that the atomic ratio of Si, Fe, Al and O is about 76.6:7.1:5.9:10.4. Si and Fe are enriched in different local areas (Figure 3h), which further confirms the formation of the dual network structure composed of the corresponding product of Si and Fe_2_O_3_. 

The formation process of iron oxide can be explained as follows. In the NaOH solution, OH^−^ groups violently collide with the Al-Fe-based ribbons to form the intermediate Fe(OH)_2_ [38]. Then, this phase decomposes into Fe, Fe_3_O_4_ and H_2_. At the same time, the reduction of Fe_3_O_4_ by H_2_ happens, inducing the formation of Fe_2_O_3_ and even α-Fe in the dealloying products. The composition of the dealloyed product is affected by the concentration of the corrosive solution, the proportion of the initial element, the ratio between the reactant and the corrosive solution, etc. In a more concentrated NaOH solution (e.g., 10 M), the reaction between Al and NaOH is enhanced, producing H_2_ in a short period. Most of the Fe_3_O_4_ is reduced and thus α-Fe will dominate in the reaction products. While in NaOH solutions with appropriate concentrations (e.g., 1–2 M), iron oxides will dominate in the reaction products.

**Figure 4:**
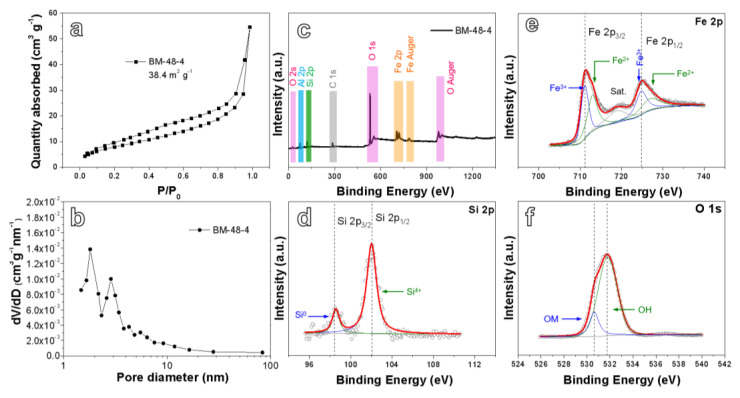
(**a**) N_2_ adsorption–desorption isotherm characteristics, (**b**) pore size distribution, (**c**) X-ray photoelectron spectroscopy (XPS) survey spectrum, and XPS spectra of (**d**) Si 2p, (**e**) Fe 2p and (**f**) O 1s for the BM-48-4.

Nitrogen adsorption–desorption isotherm and pore diameter distribution curves are displayed in Figure 4a,b, respectively. The results show that the specific surface area of BM-48-4 (38.4 m^2^ g^−1^) is higher than that of BM-24-4 (23.9 m^2^ g^−1^) and BM-72-4 (11.2 m^2^ g^−1^). A large number of mesopores less than 10 nm exist in the product (Figure 4b), which can cushion the volume expansion of the material and shorten the Li–ion diffusion distance. Based on the above analysis, BM-48-4 is expected to possess relatively good electrochemical performance [39]. The surface elements and valence states of BM-48-4 were analyzed by XPS. The full XPS spectrum shown in Figure 4c shows that elements Si, Fe, Al, O and C exist in BM-48-4 without other impure substances. The appearance of Al 2p spectra stems from un-dealloyed residual Al elements. The Si 2p spectrum in Figure 4d reveals two typical peaks of Si located at 98.3 and 102.1 eV, relating to Si^0^ and Si^4+^, respectively. This indicates that slight oxidation occurs in the outermost layer of Si [40]. Fe 2p spectrum in Figure 4e shows clear peaks concentrated at 710.8, 724.8 and 719.8 eV, which are connected to Fe 2p_3/2_, Fe 2p_1/2_ and satellite peaks, respectively. In addition, an energy difference of 14.0 eV between Fe 2p_3/2_ and Fe 2p_1/2_ can be obtained, demonstrating the generation of Fe_2_O_3_ [41,42]. The O 1s spectrum shown in Figure 4f can be divided into two peaks, the peak at 531.8 eV is from hydroxyl (OH bond, possibly from residual sodium hydroxide), and the wide peak centered at 530.8 eV can be attributed to the peak of metal bond in oxide, namely Fe-O bond (OM bond) in Fe_2_O_3_ [43]. 

**Figure 5:**
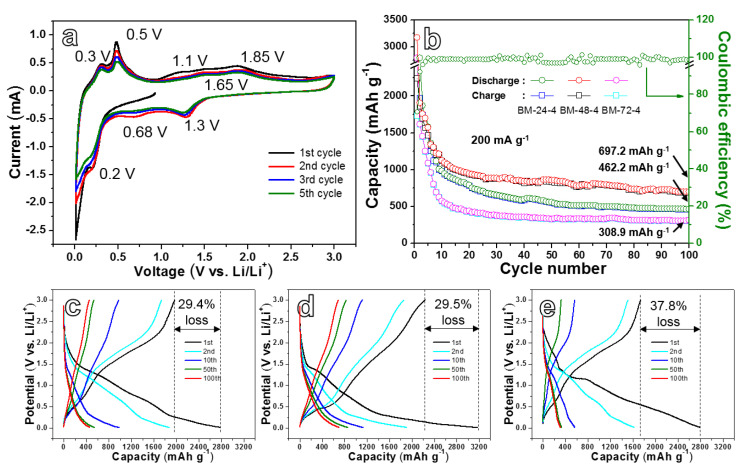
(**a**) Cyclic voltammetry (CV) curves of the BM-48-4 electrode detected at 0.1 mV s^−1^, (**b**) cyclic performances of BM-24-4, BM-48-4, BM-72-4 anodes at 200 mA g^−1^, galvanostatic charge–discharge (GCD) profiles of the Si/Fe_2_O_3_ electrodes recorded under 200 mA g^−1^: (**c**) BM-24-4, (**d**) BM-48-4 and (**e**) BM-72-4.

To study the charging/discharging reaction mechanism of Si/Fe_2_O_3_ anode in detail, a CV test was performed towards the BM-48-4 electrode with the scanning speed of 0.1 mV s^−1^ in the range of 0.01~3.0 V, as presented in Figure 5a. In the initial reduction process, an apparent peak at 0.2 V and a steep peak appeared at 0.01~0.15 V can be found, relating to the creation of amorphous Li_x_Si from lithiation of crystal Si and the generation of solid electrolyte interphase (SEI) film. Two obvious peaks of 0.3 V and 0.5 V correspond to delithiation reaction from Li_x_Si to Si [44,45] in the first anode scan. The peak appearing at about 1.1 V corresponds to the reaction between the surface oxygenic functional groups and Li^+^ [46]. A relatively wide peak at 1.85 V is in connection with the oxidation of metal Fe to Fe^2+^/Fe^3+^ and decomposition of Li_2_O. From the second cycle, two peaks can be found at 1.3 V and 0.68 V in the cathode reaction, which are believed to the multi-step electrochemical reaction from Fe_2_O_3_ to Fe (Fe_2_O_3_→Li_x_Fe_2_O_3_→Li_2_Fe_2_O_3_→Fe) [47]. While the broad peak in the anode reaction is decomposed into two peaks at 1.65 V and 1.85 V, relating to the transformation from Fe^0^ to Fe^2+^ and from Fe^2+^ to Fe^3+^, respectively. In addition, the CV curves in different cycles reflect relatively good reversibility [48]. As the number of cycles increases, the closure area of CV curves decreases slightly, showing that the BM-48-4 composite possesses acceptable cyclic stability. Although there have been a lot of studies on the application of Fe_2_O_3_ anode in LIBs, there is some controversy on its electrochemical reaction mechanism, and opinions have not been unified yet. A lot of detection and analysis is still needed in the future.

Figure 5b reveals the cycling properties of the three anodes during cycling at 200 mA g^−1^. The specific capacities of the first discharge/charge of the BM-24-4, BM-48-4 and BM-72-4 electrodes are 2787.2/1969.4, 3167.7/2234.8 and 1732.4/2784.3 mAh g^−1^, respectively. The capacity drops quickly in the initial eight cycles. After 100 cycles, the reversible capacity of the three electrodes tends to be stable, maintaining at 462.2, 697.2 and 308.9 mAh g^−1^, respectively. The BM-48-4 electrode shows the best cyclic stability among the three materials. The initial cycle coulomb efficiency of BM-48-4 is 70.5%. After five cycles, the coulomb efficiency approximates to 99.8% and around at 100% during the following cycles. Figure 5c–e displays the galvanostatic charge/discharge curves of BM-24-4, BM-48-4 and BM-72-4 during cycling under 200 mA g^−1^. Taking BM-48-4 as an example, the first discharge curve presents three platforms, which are 1.2~1.5, 0.6~1.0 and 0.01~0.2 V, respectively. While in the first charging stage, two long platforms of 0.2~0.7 and 1.1~2.0 V are found, according to the CV results. The capacity loss of the first cycle of BM-48-4 is about 29.5%, which is close to that of BM-24-4 (29.4%) and much lower than that of BM-72-4 (37.8%). With the increase in cycle number, the profile gradually moves to the left, demonstrating a slight capacity decline after several cycles. The charge/discharge profiles of BM-48-4 for the 50th and 100th cycles are close, revealing good cycling stability of the anode at the later stage of cycling. BM-24-4 and BM-72-4 electrodes show relatively low reversible capacity after cycling for 100 cycles. The huge capacity decay is induced by the serious volume variation during the cycling process, the crushing of active Si particles, the cracking of nanosheets structure, the creation of over-thick SEI film and the failure of electron and ion transport channels during the cycling process [49]. The difference in the electrochemical performance of a material in different tests in this work may be caused by the local non-uniformity of a material.

**Figure 6:**
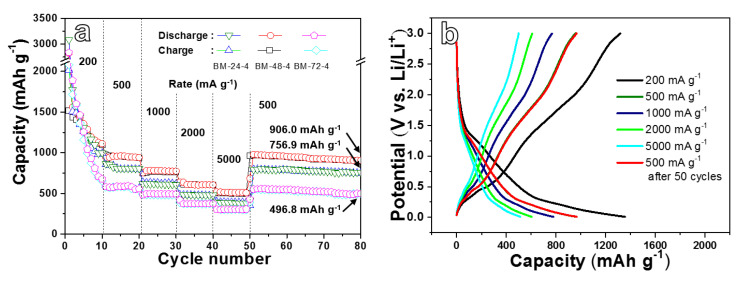
(**a**) Rate performance of BM-24-4, BM-48-4 and BM-72-4 anodes, (**b**) galvanostatic charge–discharge (GCD) curves of BM-48-4 anode at different current densities.

The rate performance of three electrode materials were tested at different current densities in the range of 200 to 5000 mA g^−1^, as presented in Figure 6a. The BM-48-4 electrode delivers the reversible capacity of 1356.5, 963.1, 779.9, 604.3 and 512.2 mAh g^−1^ under 200, 500, 1000, 2000 and 5000 mA g^−1^, respectively. At each current density, the BM-48-4 electrode shows the best specific capacity in the three anodes. When the current density recovers to 500 mA g^−1^, the BM-48-4 anode delivers a reversible capacity of 906 mAh g^−1^ after 30 cycles, which is extremely higher than the other two anodes, revealing a relatively good rate property of BM-48-4 in three electrodes. Figure 6b presents the charge and discharge curves of the BM-48-4 electrode at different current densities. It shows that with the enhancement in current density, the curve gradually shifts to the left, that is, the capacity of the electrode gradually reduces. Moreover, when the current density recovers from high current density to 500 mA g^−1^, the constant current charge/discharge profile almost coincides with the original curve tested at the same current density. All tests uncover that the BM-48-4 electrode possesses the best rate performance.

**Figure 7:**
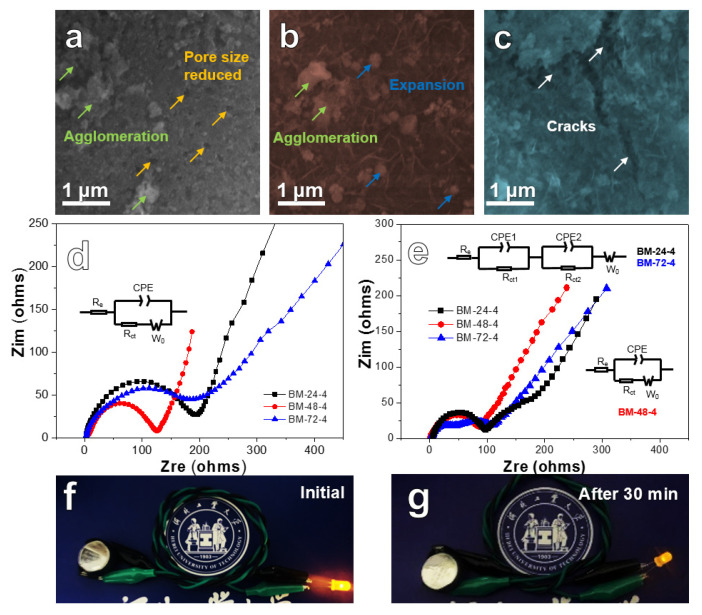
SEM images of the experimental anodes after cycling at 200 mA g^−1^ for 100 cycles: (**a**) BM-24-4; (**b**) BM-48-4 h; (**c**) BM-72-4; (**d**,**e**) Nyquist plots for BM-24-4, BM-48-4 and BM-72-4 composite anodes: (**d**) fresh and (**e**) after 100 cycles; (**f**,**g**) digital photographs of an LED bulb propelled by a Si/Fe_2_O_3_ battery: (**f**) initial and (**g**) after 30 min.

Figure 7a–c presents SEM images of three Si/Fe_2_O_3_ anodes after cycling at 200 mA g^−1^ for 100 cycles. After a long cycle, the pore size and porosity of BM-24-4 (Figure 7a) reduces, and the nanoparticles reunite to agglomeration, which is not conducive to ion transport. The BM-48-4 material (Figure 7b) can maintain the original structure of nanosheets and nanoparticles (with local agglomeration), presenting a relatively good structural stability. It is observed from Figure 7c that the surface of BM-72-4 is rough, and there are large cracks and some local aggregation. The above phenomenon reveals that BM-48-4 best preserves the original structure, which guarantees its good cyclic stability. Figure 7d,e shows the EIS data of Si/Fe_2_O_3_ anodes before and after 100 cycles. The EIS is composed of the high and medium frequency region (concave semicircle) caused by charge transfer resistance and the low-frequency region (slash) because of ion diffusion [50]. The minimum semicircle of BM-48-4 shows that the transfer resistance of the anode is significantly inferior to that of the BM-24-4 and BM-72-4 electrodes (Figure 7d). The inclination of the BM-48-4 electrode is higher than that of the BM-24-4 and BM-72-4 electrodes, indicating that the diffusion resistance of the BM-48-4 electrode is smaller. After 100 cycles, the diffusion resistance of three anodes is similar while the BM-48-4 electrode remains the smallest transfer resistance in three anodes (Figure 7e). Figure 7f shows a digital photo of a yellow light emitting diode (LED) bulb powered by an as-assembled half-cell. After 30 minutes, the LED bulb was less bright (Figure 7g) than it was when it started, but it still works, showing its good potential in practical applications.

Table 1 [14,17,19,20,34,44,51] compares the electrochemical properties of the electrode materials currently studied with those previously reported. The Li storage performance of the as-synthesized Si/Fe_2_O_3_ anode is better than that of most listed Si-based composites, and its excellent electrochemical performance is mainly attributed to the following points. Firstly, Si/Fe_2_O_3_ electrodes with a high specific surface area may provide a large area of contact and interaction between the active material and the electrolyte. Secondly, the three-dimensional porous network structure with interconnected ligaments can enhance ionic mobility and permeability. Moreover, ample pores can effectively alleviate the volume expansion of Si. In summary, Si/Fe_2_O_3_ material synthesized from natural ferrosilicon ores in this study possesses immense potential as an anode for LIBs application. Furthermore, the study also offers a new idea for the synthesis of low-cost Si-based electrodes and opens a new direction for the application of ferrosilicon ores in a brand-new field other than deoxidizing agents for steel manufacturing.

## 4. Conclusions

Porous Si/Fe_2_O_3_ dual network material was synthesized through the melt-spinning, ball-milling and dealloying process by utilizing natural ferrosilicon ores. The as-obtained Si/Fe_2_O_3_ anode displays a dual network structure. Due to this special porous structure, the BM-48-4 anode uncovers good Li storage property, delivering a good reversible capacity of 697.2 mAh g^−1^ after cycling at 200 mA g^−1^ for 100 cycles, which shows its great potential as LIBs anode. In addition, we also offer a new idea for the synthesis of porous Si-based electrodes and open a new direction for the application of ferrosilicon ores in a brand-new field.

## Figures and Tables

**Table 1:** Comparison of electrochemical properties of different Si-based materials as Lithium–ion battery (LIBs) anodes.

Si-Based Electrodes	Current Density (mA g^−1^)	Cycle Number	Reversible Capacity (mAh g^−1^)	Reference
Si@NiAl-LDH	50	60	534	[17]
Si@rGO	100	100	450	[44]
AC < nc-Si > AC	100	100	492	[51]
Si-TiO_2_	100	200	510	[34]
Si_FS_/G@C	100	100	730	[20]
Si/SiO_2_-OMC	200	100	958	[14]
Si/NC	200	100	459.2	[19]
Si/Fe_2_O_3_	200	100	697.2	This work
